# Complete Genome Sequence of *Aquiluna* sp. Strain KACHI24, Isolated from River Surface Water

**DOI:** 10.1128/mra.00858-22

**Published:** 2022-09-19

**Authors:** Yusuke Ogata, Keiji Watanabe, Shusuke Takemine, Kaoru Kaida, Maki Tanokura, Wataru Suda

**Affiliations:** a RIKEN Center for Integrative Medical Sciences, Tsurumi-ku, Yokohama, Kanagawa, Japan; b Center for Environmental Science in Saitama, Kazo, Saitama, Japan; University of Southern California

## Abstract

The globally distributed bacterioplankton of the genus *Aquiluna* belong to the tribe Luna1-A1. Here, we report the complete genome sequence of *Aquiluna* sp. strain KACHI24, which was isolated from river surface water in Japan.

## ANNOUNCEMENT

Bacterioplankton belonging to the genus *Aquiluna* bacteria have worldwide distribution, including freshwater and marine environments (1–3). However, the lack of archived and reported sequences belonging to this genus is mainly a result of limited success in isolating a pure culture (2, 3). Therefore, accessing the genomic information of *Aquiluna* bacteria is important for the estimation of physiochemical and ecological insights into the genus.

Here, we report the complete genome sequence of an *Aquiluna* sp. strain, KACHI24 (JCM 32205), isolated from a sample of river surface water collected from the Ichino River (Kachi Bridge), Yoshimi, Saitama, Japan (36°01′08.8″N, 139°28′23.9″E) on 2 July 2013. The sample was filtered through a disposable syringe equipped with a 0.7-μm particle retention glass fiber filter (Puradisc 25 GF/F disposable filter device; Whatman, Springfield Mill, UK). The filtrate was then spread onto modified Reasoner’s 2A (MR2A) agar plates and incubated at 25°C for 2 to 5 days (4). One bacterial colony was picked, inoculated into sterilized MR2A liquid medium (pH 7.2), and incubated at 25°C for 2 days with reciprocal shaking (120 rpm). The pure strain cell suspension was maintained in MR2A broth supplemented with 20% (wt/vol) glycerol stocks at −80°C for preservation. Glycerol stock was inoculated and cultivated in MR2A liquid medium, and the cells were harvested by centrifugation for genomic DNA extraction.

Genomic DNA of strain KACHI24 was extracted using an enzymatic lysis and phenol-chloroform-isoamyl alcohol method as previously described (5). Whole-genome sequencing of this strain was performed using the Sequel II platform (Pacific Biosciences of California, Inc., Menlo Park, CA, USA), and the library was prepared using the SMRTbell template prep kit v.2.0 (PacBio), with DNA shearing using the g-TUBE device (Covaris, LLC, Woburn, MA, USA) with a target length of 10 to 15 kb. The PacBio reads were converted to consensus reads (CCS reads) using the software CCS v.6.2.0 (https://github.com/PacificBiosciences/ccs). The CCS reads were assembled using the assembler Canu v.2.1.1 with specified parameters (minReadLength = 2200 minOverlapLength = 2200) (6), and the obtained genome sequence was annotated using DFAST v.1.6.0 (https://dfast.nig.ac.jp) (7). We also checked the circularization of the generated genome sequence to remap the CCS reads using Minimap2 v.2.24-r1122 (8). Default parameters were used for all software analysis, unless otherwise specified, and information on the obtained reads and generated genome sequences is described in Table 1.

**TABLE 1:** Information on the reads and contig obtained for *Aquiluna* sp. strain KACHI24

Parameter	Data
Data for quality-checked Sequel reads	
No. of reads	27,153
Total no. of bases	389,909,142
*N*_50_ (bp)	14,219
Total no. of contigs	1
BioProject accession no.	PRJDB14121
BioSample accession no.	SAMD00518422
Sequence Read Archive (SRA) accession no.	DRR396571
Genome size (bp)	1,459,348
GC content (%)	53.8
GenBank/ENA/DDBJ accession no.	AP026677

Based on the annotation results of the genome sequence of strain KACHI24, we identified the presence of a gene predicted to encode polyphosphate kinase (*ppk*), which is associated with the intracellular accumulation of polyphosphate and a putative xanthorhodopsin-like protein-encoding gene, which is related to a putative light-driven, proton-pumping rhodopsin (9). Furthermore, this strain also contained putative alanine dehydrogenase and glycine dehydrogenase-like protein-encoding genes, which are associated with the process of ammonification.

### Data availability.

The chromosome sequence and reads of strain KACHI24 were deposited at GenBank/ENA/DDBJ, under the accession no. AP026677, which is linked to the BioProject accession no. PRJDB14121, the BioSample accession no. SAMD00518422, and the DDBJ Sequence Read Archive accession no. DRR396571.
